# QTL Analysis Identifies a Modifier Locus of Aganglionosis in the Rat Model of Hirschsprung Disease Carrying *Ednrb^sl^* Mutations

**DOI:** 10.1371/journal.pone.0027902

**Published:** 2011-11-22

**Authors:** Ruihua Dang, Daisuke Torigoe, Nobuya Sasaki, Takashi Agui

**Affiliations:** Laboratory of Laboratory Animal Science and Medicine, Department of Disease Control, Graduate School of Veterinary Medicine, Hokkaido University, Hokkaido, Japan; National Cancer Institute, United States of America

## Abstract

Hirschsprung disease (HSCR) exhibits complex genetics with incomplete penetrance and variable severity thought to result as a consequence of multiple gene interactions that modulate the ability of enteric neural crest cells to populate the developing gut. As reported previously, when the same null mutation of the *Ednrb* gene, *Ednrb^sl^*, was introgressed into the F344 strain, almost 60% of F344-*Ednrb^sl/sl^* pups did not show any symptoms of aganglionosis, appearing healthy and normally fertile. These findings strongly suggested that the severity of HSCR was affected by strain-specific genetic factor (s). In this study, the genetic basis of such large strain differences in the severity of aganglionosis in the rat model was studied by whole-genome scanning for quantitative trait loci (QTLs) using an intercross of (AGH-*Ednrb^sl^*×F344-*Ednrb^sl^*) F_1_ with the varying severity of aganglionosis. Genome linkage analysis identified one significant QTL on chromosome 2 for the severity of aganglionosis. Our QTL analyses using rat models of HSCR revealed that multiple genetic factors regulated the severity of aganglionosis. Moreover, a known HSCR susceptibility gene, *Gdnf*, was found in QTL that suggested a novel non-coding sequence mutation in GDNF that modifies the penetrance and severity of the aganglionosis phenotype in *EDNRB*-deficient rats. A further identification and analysis of responsible genes located on the identified QTL could lead to the richer understanding of the genetic basis of HSCR development.

## Introduction

Hirschsprung disease (HSCR) is a congenital malformation characterized by the absence of intramural ganglion cells along variable lengths of the distal gut. Due to the lack of ganglia, the stool cannot be passed through the colon, and the bowel wall is dilated [1]–[4]. The disorder is classified into short-segment (S-HSCR, 80%), long-segment (L-HSCR, 15%), or total colonic aganglionosis (TCA, 5%) [5]. HSCR is observed in about 1/5000 live birth and is more frequent in males than in females (4∶1), a difference most prominent in S-HSCR [6]. Several genes have been implicated in the development of HSCR, including the *RET* proto-oncogene [7]–[9], endothelin receptor B gene (*EDNRB*) [10]–[17], endothelin-3 gene (*EDN3*) [18], [19], glial-cell-line-derived neurotrophic factor (*GDNF*) [20]–[22], *SOX10* [23], [24], *NRTN* [25], *ECE1* [26], *ZFHX1B* [27], *PHOX2B* [28], *KIAA1279* [29], *TCF4* [26]. However, mutations in these genes explain only a minority of cases and the vast majority (80%) of HSCR heritability remains unknown [30]. HSCR displays a highly variation in penetrance and phenotypes by gender, familial incidence, segment length of aganglionosis and associated phenotypes. The variable penetrance and expressivity of this disease are attributed to the complex genetic interactions between the known susceptibility loci and undiscovered susceptibility or modifier loci in the genetic background that modulates the ability of enteric neural crest cells to populate the developing gut [31].

Many researchers have used inbred models to search the unknown susceptibility or modifier genes of aganglionosis [31], [32]. Moreover, mouse models, in which genetic background and input alleles can be controlled in genome-wide and candidate gene approaches, are a strong tool to identify the novel genetic factors or modifiers that influence the variable penetrance and inheritance patterns of complex diseases like HSCR. Spotting lethal (*sl*) is a spontaneous null mutation that has a 301 bp deletion in the rat *Ednrb* gene that results in the absence of a functional receptor protein [33]. In the previous study, we established an AGH-*Ednrb^sl^* [34] inbred strain carrying the *sl* mutation, further, introgressed this mutation into LEH and F344 strains to produce two congenic strains: LEH-*Ednrb^sl^* and F344-*Ednrb^sl^* [34]. In AGH-*Ednrb^sl/sl^* rats, only 20% of pups survived until weaning; whereas in F344-*Ednrb^sl/sl^* rats, 100% of pups survived to weaning. Interestingly, almost 60% of F344-*Ednrb^sl/sl^* pups did not show any symptoms of aganglionosis, appearing healthy and normally fertile and showing normal body weight gain. Thus, we concluded that variation in the penetrance and survival was attributable to distinct differences in the severity of aganglionosis, and resistant genes in the genetic background of F344 significantly modulated the severity of the aganglionosis phenotype.

This study focuses on the variation in aganglionosis between individual *Ednrb^sl^*-mutated rats and uses this variation to identify modifiers that are influencing the aganglionosis aspect of the phenotype. These studies have been facilitated by the ability to control genetic background in inbred lines of *Ednrb^sl^* rats that are not possible in patient studies.

## Results

### Evaluation of aganglionosis as a quantitative trait in F_2_ *Ednrb^sl/sl^* rats

**Figure 1:**
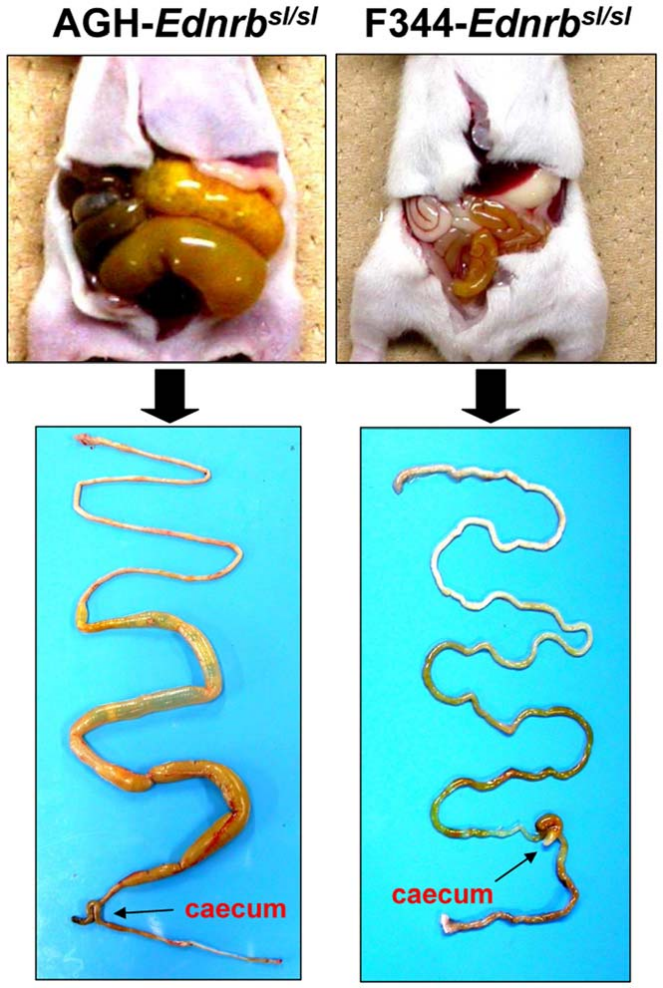
Comparison of the expressivity of aganglionosis. 14-day-old AGH-*Ednrb^sl/sl^* rats (left) show severe symptoms of aganglionosis, but not in F344-*Ednrb^sl/sl^* rats (right).

**Figure 2:**
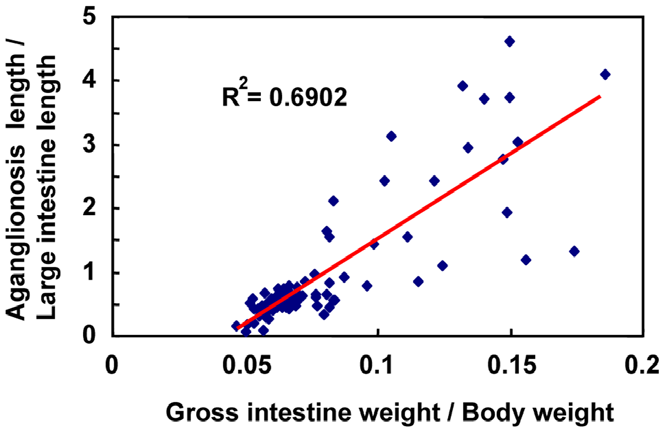
Correlation analysis between the severity of aganglionosis and the ratio of gross intestine weight. Correlation analysis between the severity of aganglionosis (aganglionosis length/large intestine length) and the ratio of gross intestine weight (gross intestine weight/body weight) shows a high correlation between the two traits.

Homozygous *Ednrb^sl/sl^* rats showed aganglionosis phenotypes. In our previous study, we found that when the *sl* mutation was introgressed into the F344 strain, the phenotype of aganglionosis was strongly modified [34]. As shown in Fig. 1, AGH-*Ednrb^sl/sl^* rats at postnatal 14 day exhibited abnormal dilation of the intestines resulting from the absence of ganglion cells in a long segment beyond caecum. In contrast, in F344-*Ednrb^sl/sl^* pups at postnatal 14 day, an enlarged small intestinal phenotype (mega small intestine) was not found. We have confirmed that the variation in the expressivity of this disease between these two strains was caused from the extent of aganglionosis by whole-mount acetylcholinesterase (AChE) staining [34]. We used the same method to establish the range of phenotypes among the F_2_ (AGH×F344) *Ednrb^sl/sl^* progenies. The F_2_ animals (n = 410) were produced by heterozygotes mating between AGH and F344 strains and then 96 *Ednrb^sl/sl^* pups were selected to phenotype based on the difference in skin pigmentation pattern between homologous mutants and other genotype rats or genotyping for the *Ednrb^sl^* mutation (for albino pups). The number of *Ednrb^sl/sl^* pups was consistent with the anticipated 25% transmission ratio. Microscopic examination of *Ednrb^sl/sl^* intestines stained by AChE was used to appraise the length of aganglionosis gut, then the extent of aganglionosis was calculated as a ratio of length of the aganglionosis intestine to the length of the entire large intestine used as a quantitative trait in individual animals. We also recorded the gross intestine weight and body weight of pups at postnatal 14 day, and to fully capture the difference between sick and healthy ones, the ratio of gross intestine weight (gross intestine weight/body weight) was calculated, which demonstrates the expressivity of megacolon directly. We found that there was a high correlation between the aganglionosis extent and the ratio of gross intestine weight in F_2_ populations (Fig. 2). This showed that the ratio of aganglionosis extent is appropriate as a quantitative trait for the severity of aganglionosis. The specificity and sensitivity of the extent of aganglionosis as a quantitative trait were confirmed by following experiments using MapManager QTXb.

**Figure 3:**
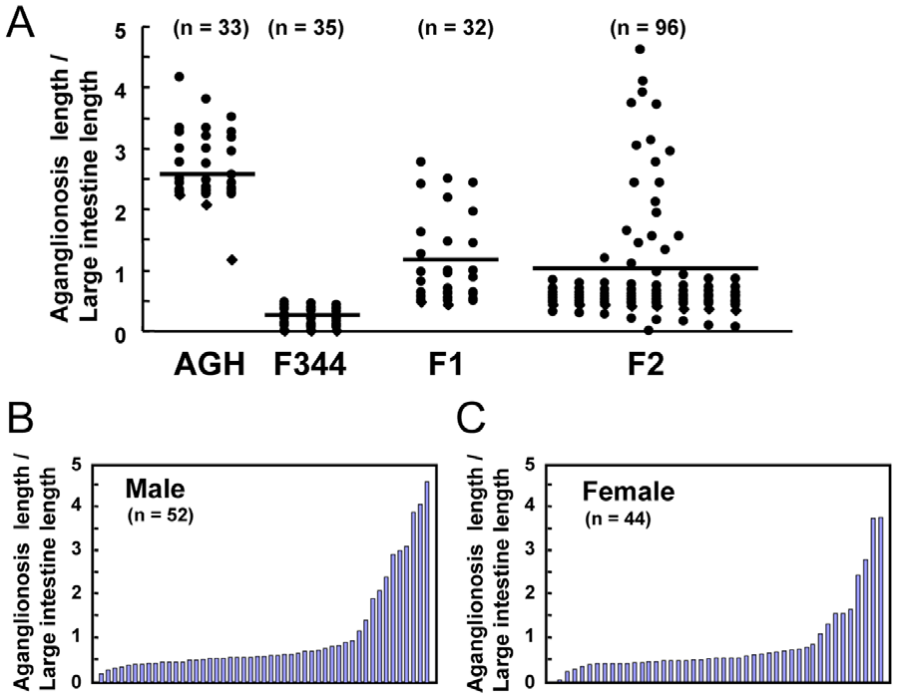
The range of the aganglionosis extent. (A) The range of the aganglionosis extent in 14-day old pups from AGH-*Ednrb^sl/sl^*, F344-*Ednrb^sl/sl^*, F_1_, and F_2_. Mean values are indicated by horizontal lines. Distribution of the severity of aganglionosis in male (B) and female (C) F_2_ progenies.

The range of the aganglionosis extent for each progeny is presented as black characters in Fig. 3A, which was fairly scattered for F_2_ intercross progenies, while that of the AGH and F344 progenies tended to fall on one of the two extremes. The mean ratio of the F_2_ progenies (0.95 in ratio of aganglionosis extent) composed of each homozygote of AGH and F344, and the heterozygotes were nearly the same as that of the F_1_ progenies (1.08 in ratio of aganglionosis extent).

In Fig. 3B and 3C, individual traits of the male and female F_2_ progenies are arranged by size of the ratio of aganglionosis extent. The trait-value graphs in both males and females showed similar gentle curves, which suggests that the mild aganglionosis extent in F344- *Ednrb^sl/sl^* rats are under the control of polygenic inheritance.

### QTL analysis identifies modifiers of aganglionosis severity in *Ednrb^sl/sl^* rats

**Figure 4:**
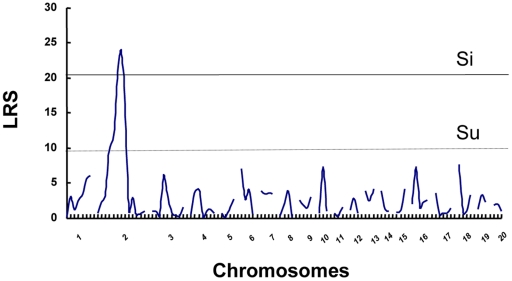
Result of interval mapping scans by MapManager QTXb in F_2_ rats. Analyses of linkage of aganglionosis severity in F_2_ populations to chromosomal loci were performed using the MapManager QTXb20 software. Recombination frequencies (%) were converted into genetic distance (centiMorgan; cM) using the Kosambi map function, in which linkage data are provided as likelihood ratio statistic (LRS) scores. Genome-wide significance thresholds were calculated in terms of LRS by carrying out permutation tests for 500 permutations. The thresholds for suggestive (Su), significant (Si) linkages are indicated in dotted and thin lines, respectively. LRS, likelihood ratio statistic.

**Figure 5:**
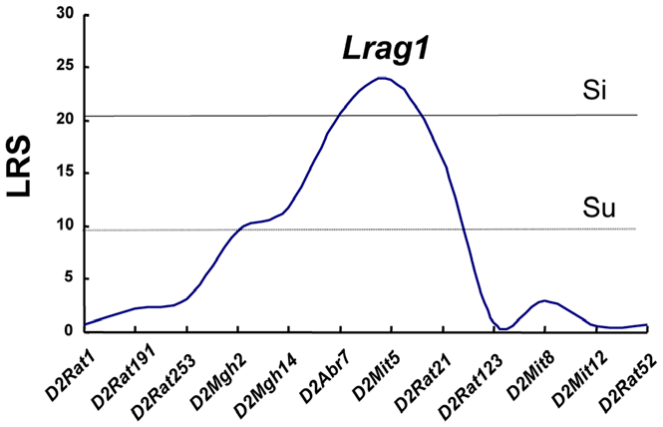
Details of suggestive and significant linkages in QTL analysis of the severity of aganglionosis. The QTL on chromosomes 2 (*Lrag1*) showed a significant linkage to aganglionosis severity, respectively. The dotted and thin lines represent suggestive (Su) and significant (Si) thresholds, respectively. The microsatellite markers used for determining genotypes of F_2_ rats are presented along the X-axis. LRS, likelihood ratio statistic.

Final results of interval mapping were considered suggestive, significant, or highly significant linkages when the threshold likelihood ratio statistics (LRS) were 9.9, 20.2, and 30.4, respectively. As shown in Fig. 4, the highest linkage over the significant level (LRS>20.2) appeared on Chr 2. The maximum LRS score was 23.9 on Chr 2. Linkage details were shown in Fig. 5. The locus at the *D2Mit5* marker position, showing the highest linkage to the severity of aganglionosis (LRS = 23.9), was designated ‘*Lrag1 (Locus of resistance to aganglionosis 1)*’. The epistatic interaction between markers also was searched by MapManager QTXb, but no significant interaction was found.

### Allele effects of *Ednrb^sl/sl^* modifier loci

**Figure 6:**
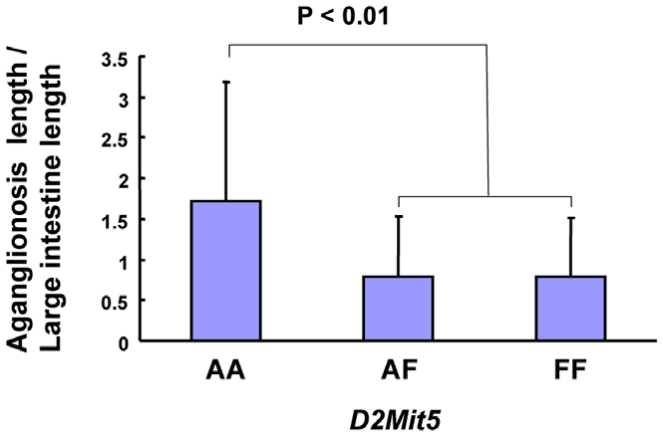
Effect of alleles at *Ednrb^sl/sl^* modifier loci on the severity of aganglionosis. Genotypes from the total F_2_ population obtained from the marker closest to the modifier were used to assess the effects of individual loci on the severity of phenotype. The mean of aganglionosis severity (aganglionosis length/large intestine length) is plotted for each genotype class to show the relation of the number of AGH or F344 alleles and the extent of aganglionosis for this locus. Markers used to generate genotype information are listed beneath the plot. Genotype groups are defined as AGH/AGH (AA), AGH/F344 (AF) and F344/F344 (FF).

Modifier loci either can increase susceptibility and severity of phenotype or can act protectively to confer resistance to disease in the face of a predisposing mutation [35]. To assess the effects of individual *Ednrb^sl/sl^* modifiers on the severity of aganglionosis, we evaluated complete genotype information in the total F_2_ distribution. F344 alleles at modifier locus on chromosome 2 decreased the extent of gut length affected by aganglionosis (Fig. 6). The allele effect observed was approximately dominant, with heterozygotes exhibiting phenotypes equal to the phenotypes of the homozygous animals of F344 alleles.

### Identification of candidate genes in chromosome 2

By bioinformatics methods combining genome annotation with literature searches, some biologically relevant genes within modifier intervals have been identified successfully [36]. We used the positions of the closest markers flanking the peak on Chr 2 to define the boundaries of this interval on the rat genome assembly and searched for genes that might be involved in development of enteric neural system based on their expression profiles in the literature and public databases. More than 30 genes within this interval were identified by NCBI. This listing of candidates was narrowed to include only those genes associated with cell migration, the development of enteric nervous system based on information in the PosMed and in the Gene Expression Database (Table 1). Within these genes, two highly relevant candidates were identified based on their documented expression in the developing gut. These included *Gdnf* and *Rai14* genes. *Rai14* is expressed early in the neural tube of 9.5-day mouse embryo and maintained in intestines. *Gdnf* encodes a highly conserved neurotrophic factor, which promotes the survival of many types of neurons. *Gdnf*-null mice showed a complete absence of the enteric nervous system, ureters, and kidneys [37]. *GDNF* is established to be important in the development of the enteric nervous system and Hirschsprung disease. So it is a logical and possible candidate modifier. We sequenced the coding region of *Gdnf*, but failed to find a difference between the two rat strains. Subsequently, we also compared the expression level of *Gdnf* mRNA of the whole intestine tissue from wildtype and heterozygous AGH and F344 rats in embryonic day 15.5 by RT-PCR. However, no difference was found (data not shown).

**Table 1:** List of candidate genes for *Lrag1*.

*Lrag1 candidates on Chr 2*		
Gene symbol	Gene description	Mbp
***D2Arb7***	Flanking marker	57.2
***Gdnf***	Glial cell line-derived neurotrophic factor	57.4
*Rai14*	Retinoic acid induced 14	59.9
*Zfr*	Zinc finger RNA-binding protein	61.6
*Mtmr12*	Myotubularin-related protein 12	61.6
*Mtmr1*	Myotubularin related protein 1	61.7
*Golph3*	Golgi phosphoprotein 3	61.8
*Pdzd2*	PDZ domain containing 2	61.8
***D2Mit5***	Flanking marker	66.7

## Discussion

The enteric nervous system (ENS) mostly derives from migratory vagal neural crest cells. A minority of the foregut ENS also arise from migratory anterior trunk neural crest cells of the posterior vagal region [4]. Neural crest cells enter the foregut at embryonic day 9–9.5 in mice, in this time they are termed enteric neural crest-derived cells (ENCCs) [4]. These progenitor cells of enteric nervous system migrate in a rostral to caudal direction to sequentially colonize the foregut, midgut, and last the hindgut, which is complete by embryonic day 15 [2], [4]. Neural crest cells from sacral levels of the neural tube also colonize the gut, where they contribute to only a small fraction of enteric neurons and glia in the distal midgut and hindgut [2], [4]. ENCCs proliferate actively to expand the relatively small pool of progenitors and then differentiate into phenotypically distinct neuronal subtypes and glia. The multi-step, complex nature of ENS ontogeny suggests that it is vulnerable to alterations in the function or expression of many genes as well as changes in the environment. When this progress is disturbed, a congenital gut motility disorder, HSCR occurs, which is characterized by an absence of enteric neurons in terminal regions of the gut. HSCR is a complex disease manifesting with low, sex-dependent penetrance and variability in the length of the aganglionic segment [32].

In human with HSCR, the genetic interaction between mutations in *RET* and *EDNRB* was found in an association study conducted on Mennonite family with the W276C mutations in the *EDNRB* [38]. The combination of these two genotypes increased the penetrance of the W276C mutation and therefore the risk of disease. Genetic interaction between *RET* and *EDNRB* pathways has also been demonstrated in mice [38]–[40]. In mice, heterozygosity for two known mutant HSCR genes, *RET^+/−^* and *Ednrb^sl^*, or *RET^+/−^* and *Ednrb^s^* genes, had no intestinal aganglionosis, whereas *RET^+/−^* mice with the homozygous *Ednrb^s^* or heterozygous *Ednrb^s^*/*Ednrb^sl^* mutations showed megacolon [38], [40]. Thus, the synergistic effects of multiple mutations in HSCR-associated genes can influence disease penetrance and expressivity. The mechanisms underlying these interaction may help to explain the complexity of the HSCR phenotype and resolve puzzling genetic observations, such as variations in penetrance and severity of aganglionosis between family members carrying equivalent mutations in HSCR genes [41]. However, many susceptibility genes or modifier genes or interaction between them remain unknown. Animal models have greatly helped us to understand HSCR genetics and embryologic events that construct the ENS. Several susceptibility genes of HSCR are initially identified in mice that are later found to be altered in human HSCR patients [42], [43].

In this study, we used quantitative trait locus (QTL) mapping to detect the genetic loci that contribute to differences in phenotypic variation of aganglionosis extent between F344 and AGH strains with the same null mutations. Using this comprehensive approach, we have successfully identified a modifier locus of *Ednrb^sl/sl^* on rat chromosomes 2. This locus contains a known aganglionsis susceptibility gene, *GDNF*. The *GDNF* ligand activates the *RET* receptor through the assembly of a multiprotein complex, including the *GDNF* family receptor *alpha1* (*GFRalpha1*) molecule, which have important functions in the development and maintenance of sensory, enteric, sympathetic and parasympathetic neurons and a variety of non-neural tissues [44]. The genetic interaction between mutations in *RET* and *EDNRB* has been well described in human patients and confirmed in mice [38]–[40]. So it is possible that there was an interaction between *Gdnf* ligand and *Ednrb*. Though we failed to find the sequence difference of coding region in *Gdnf* gene between both rat strains, we cannot completely exclude the possibility that *Gdnf* is a responsible gene because the non-coding regulatory region of *Gdnf* remains unknown which could affect the *Gdnf* expression in a specific timing that is important for the ENS development. Such case has been found in human with HSCR that a non-coding *RET* variant within a conserved enhancer-like sequence in intron 1 is significantly associated with HSCR susceptibility [45]. We only investigated the expression level of *Gdnf* in embryonic day 15.5. It remains unknown whether there is difference in the expression level at other developmental stage of ENS. The spacial and temporal control of gene expression in the complex process of ENCCs colonization of the gut is very important. *Ednrb* is genetically required in the mouse for ENS development in vivo from embryo day 10 to embryo day 12.5 [46]. The interaction of *RET* and *EDNRB* signaling pathways only influenced the ENS, no impact on melanocyte, retinal choroid, and kidney development, which showed a tissue-specific interaction [40]. All these lines of evidence suggested a possibility that a non-coding variant of *Gdnf* interacting with *Ednrb* mutation in AGH strain resulted in the serious aganglionosis.

Using congenic techniques, we are currently attempting to generate rat strains that harbor QTLs from one selection line on the opposite line to investigate whether each allele has a different effect on the phenotype. At the same time, several approaches are currently being employed to identify candidate genes located on *Lrag1*. Some of these approaches include comparisons of gene expression levels of F344 and AGH rats in intestine tissues using microarray and next-generation RNA sequencing technologies. This analytical combination that includes QTL mapping and gene expression profiles has proven useful in the selection of candidate genes.

A lack of existing comprehensive information on the susceptibility genes and interaction between susceptibility loci or modifier loci contributing to HSCR disease in the genetic background makes it difficult to understand the genetic base for many cases of HSCR. However, our study localized chromosomal sites where the allelic differences in genes presented in F344 and AGH rats and strongly affected the occurrence and severity of HSCR using QTL analysis. This study provided the new evidence that Hirschsprung disease is the consequence of multiple gene interactions that modulate the ability of enteric neural crest cells to populate the developing gut.

## Materials and Methods

### Animals

Heterozygous AGH/Hkv-*Ednrb^sl^* (AGH) [34] and F344-*Ednrb^sl^* (F344) [34] rats were bred to generate F_2_ animals (n = 410), in which 96 *Ednrb^sl/sl^* pups were selected to phenotype based on the difference in skin pigmentation pattern. Namely, heterozygous AGH-*Ednrb^sl^* rats had pigmented heads, backs, and tails. In contrast, homozygous mutant rats had almost no pigmentation on their heads previously described [34]. On the other hand, since F344 is an *albino* (tyrosinase mutant) strain, *albino* F_2_ rats were genotyped to distinguish homozygote from heterozygote and wildtype by PCR. Animals were genotyped for *Ednrb^sl^* mutation using primers (F-CCTCCTGGACTAGAGGTTCC and R-ACGACTTAGAAAGCTACACT) that flank the site of the 301-base deletion. PCR products were electrophoresed in 2% agarose gels to distinguish the wild (511 bp) and mutant (210 bp) alleles. To determine the aganglionosis extent by strain, AGH (n = 33), F344 (n = 35), F_1_ (n = 32) were raised. Animals were maintained in specific pathogen-free conditions with feeding and drinking allowed *ad libitum*. All research and experimental protocols were conducted according to the Regulation for the Care and Use of Laboratory Animals of Hokkaido University and were approved by the Animal Care and Use Committee of Hokkaido University (Approval ID: No. 110226).

### Microsatellite genotyping

The genome-wide scan was performed using 96 intercross progenies. Genomic DNA was extracted from tail clips of these intercross progenies using a standard protocol and was subjected to a genome-wide scan at 10–30 Mbp resolution using 94 polymorphic microsatellite markers (Table 2). PCR primers of the markers were identified in the Rat Genomic Database of Ensembl (http://uswest.ensembl.org). Amplified samples were electrophoresed in 10% acrylamide gels, stained with ethidium bromide, and photographed under an ultraviolet lamp.

**Table 2:** Microsatellite markers used for genotyping AFF_2_ intercrossed progenies.

*Microsatellite Markers*	Position (Mbp)	*Microsatellite Markers*	Position (Mbp)	*Microsatellite Markers*	Position (Mbp)	*Microsatellite Markers*	Position (Mbp)	*Microsatellite Markers*	Position (Mbp)
*D1Rat392*	19	*D3Rat276*	18	*D6Rat165*	93	*D11rat43*	85	*D17Rat11*	30
*D1Got45*	36	*D3Rat80*	32	*D6Rat11*	115	*D12Rat58*	4	*D17Rat12*	33
*D1Mgh6*	87	*D3Rat93*	75	*D14Mgh4*	138	*D12Got26*	13	*D17Mit4*	71
*D1Rat269*	126	*D3Rat287*	98	*D7Rat31*	28	*D12Rat76*	29	*D17Rat58*	81
*D1Rat163*	163	*D3Mit4*	131	*D7Rat21*	96	*D13Rat59*	31	*D18Rat132*	25
*D1Rat159*	198	*D3Rat78*	146	*D7Rat14*	101	*D13Rat85*	72	*D18Got63*	67
*D1Rat235*	248	*D3Rat2*	164	*D7Mit16*	120	*D13Mit4*	90	*D18Rat86*	68
*D2Rat1*	9	*D3Rat1*	170	*D8Mit5*	32	*D14Got35*	29	*D18Rat6*	77
*D2Rat191*	21	*D4Rat222*	18	*D8Rat33*	73	*D14Rat12*	41	*D19RAT15*	15
*D2Rat253*	25	*D4Mgh2*	36	*D8Mgh4*	86	*D14Rat38*	99	*D19Mit9*	27
*D2Mgh2*	39	*D4Rat122*	64	*D8Rat18*	97	*D15Rat5*	22	*D19Got53*	49
*D2Mgh14*	42	*D4Rat183*	125	*D9Got27*	14	*D15Rat6*	32	*D20Mgh5*	11
*D2Arb7*	57	*D4Rat141*	151	*D9Mit3*	55	*D15Rat11*	51	*D20Got38*	39
*D2Mit5*	66	*D4Rat64*	156	*D9Rat15*	62	*D15Mgh5*	102	*D20Got47*	50
*D2Rat21*	75	*D4Rat67*	161	*D9Rat99*	87	*D16Rat78*	19		
*D2Rat123*	112	*D5Rat196*	103	*D10Rat217*	17	*D16Rat3*	45		
*D2Mit8*	148	*D5Got47*	131	*D10Rat24*	79	*D16Got63*	69		
*D2Mit12*	174	*D5Got93*	158	*D10Rat7*	105	*D16Rat55*	76		
*D2Rat52*	200	*D5Rat44*	159	*D11Mit4*	22	*D16Got90*	79		
*D3Rat57*	4	*D6Rat30*	48	*D11Rat5*	56	*17Rat2*	9		

AFF_2_: (AGH-*Ednrb^sl^*×F344-*Ednrb^sl^*) F_2_.

### Whole-mount staining

The guts from pups at postnatal day 14 were dissected as a single piece from the proximal esophagus to the distal colon. Mesenteric attachments and the pancreas were removed, and the guts were then processed for acetylcholinesterase (AChE) whole-mount staining using routine protocols to visualize enteric ganglia [31]. The extent of the gut regions affected by aganglionosis was determined by microscopic examination. The entire length of the gut and the large intestine length, as well as any aganglionic regions, were measured. The length of the aganglionic segment was divided by the whole large intestine length to yield an aganglionosis ratio.

### Linkage analysis

To identify the aganglionosis modifier loci, genotyping data and the ratio of aganglionosis extent were analyzed by MapManager QTXb [47], whereby permutation tests were done in 1-cM steps for 500 permutations to determine the suggestive, significant, or very significant levels of statistics.

### Statistical analyses

For comparison of allele effect at *Ednrb^sl^* modifier loci, the *t*-test was performed to compare the mean values for data sets.
